# Durability of pulmonary vein isolation for atrial fibrillation: a meta-analysis and systematic review

**DOI:** 10.1093/europace/euad335

**Published:** 2023-11-07

**Authors:** Teodor Serban, Diego Mannhart, Qurrat-ul-ain Abid, Andres Höchli, Sorin Lazar, Philipp Krisai, Arianna Sofia Bettelini, Sven Knecht, Michael Kühne, Christian Sticherling, Jeanne du Fay de Lavallaz, Patrick Badertscher

**Affiliations:** Department of Cardiology, University Hospital of Basel, Petersgraben 4, 4031 Basel, Switzerland; Cardiovascular Research Institute Basel, Spitalstrasse 2, 4056 Basel, Switzerland; Department of Cardiology, University Hospital of Basel, Petersgraben 4, 4031 Basel, Switzerland; Cardiovascular Research Institute Basel, Spitalstrasse 2, 4056 Basel, Switzerland; Department of Cardiology, Rush Medical Centre, Chicago, IL, USA; Department of Cardiology, Triemli Stadtspital, Zürich, Switzerland; Department of Cardiology, Cook County Health, Chicago, IL, USA; Department of Cardiology, University Hospital of Basel, Petersgraben 4, 4031 Basel, Switzerland; Cardiovascular Research Institute Basel, Spitalstrasse 2, 4056 Basel, Switzerland; Department of Cardiology, University Hospital of Basel, Petersgraben 4, 4031 Basel, Switzerland; Cardiovascular Research Institute Basel, Spitalstrasse 2, 4056 Basel, Switzerland; Department of Cardiology, University Hospital of Basel, Petersgraben 4, 4031 Basel, Switzerland; Cardiovascular Research Institute Basel, Spitalstrasse 2, 4056 Basel, Switzerland; Department of Cardiology, University Hospital of Basel, Petersgraben 4, 4031 Basel, Switzerland; Cardiovascular Research Institute Basel, Spitalstrasse 2, 4056 Basel, Switzerland; Department of Cardiology, University Hospital of Basel, Petersgraben 4, 4031 Basel, Switzerland; Cardiovascular Research Institute Basel, Spitalstrasse 2, 4056 Basel, Switzerland; Department of Cardiology, University Hospital of Basel, Petersgraben 4, 4031 Basel, Switzerland; Cardiovascular Research Institute Basel, Spitalstrasse 2, 4056 Basel, Switzerland; Department of Cardiology, University Hospital of Basel, Petersgraben 4, 4031 Basel, Switzerland; Cardiovascular Research Institute Basel, Spitalstrasse 2, 4056 Basel, Switzerland

**Keywords:** Atrial Fibrillation, Pulmonary Vein Isolation, Radiofrequency Ablation, Cryoballoon Ablation, Laser Balloon, Pulsed-Field Ablation

## Abstract

**Aims:**

Pulmonary vein isolation (PVI) plays a central role in the interventional treatment of atrial fibrillation (AF). Uncertainties remain about the durability of ablation lesions from different energy sources. We aimed to systematically review the durability of ablation lesions associated with various PVI-techniques using different energy sources for the treatment of AF.

**Methods and results:**

Structured systematic database search for articles published between January 2010 and January 2023 reporting PVI-lesion durability as evaluated in the overall cohort through repeat invasive remapping during follow-up. Studies evaluating only a proportion of the initial cohort in redo procedures were excluded. A total of 19 studies investigating 1050 patients (mean age 60 years, 31% women, time to remap 2–7 months) were included. In a pooled analysis, 99.7% of the PVs and 99.4% of patients were successfully ablated at baseline and 75.5% of the PVs remained isolated and 51% of the patients had all PVs persistently isolated at follow-up across all energy sources. In a pooled analysis of the percentages of PVs durably isolated during follow-up, the estimates of RFA were the lowest of all energy sources at 71% (95% CI 69–73, 11 studies), but comparable with cryoballoon (79%, 95%CI 74–83, 3 studies). Higher durability percentages were reported in PVs ablated with laser-balloon (84%, 95%CI 78–89, one study) and PFA (87%, 95%CI 84–90, 2 studies).

**Conclusion:**

We observed no significant difference in the durability of the ablation lesions of the four evaluated energies after adjusting for procedural and baseline populational characteristics.

## Graphical Abstract

**Graphical abstract:**
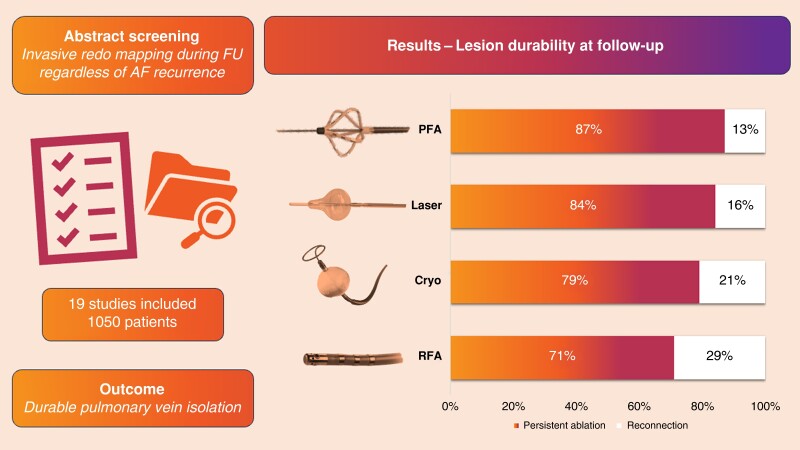

## Introduction

Several energy sources and ablation devices are currently available for pulmonary vein isolation (PVI), all considered to have similar efficacy and comparable clinical recurrence rates.^1,2^ Radiofrequency ablation (RFA)^3–17^ and cryoballoon^17–20^ are the most used and widely available techniques and have shown their efficacy and safety in large randomized clinical trials.^21–24^ However, several studies reported high rates of pulmonary vein (PV) reconnection, often associated with clinical recurrence of AF and the need for redo procedures to achieve long-term persistent PVI.^10,25–29^ Laser balloon provide the operator with the possibility of direct visualization of the ablation site, so far without providing any additional benefits regarding AF-free survival or procedure duration compared to RFA or Cryoenergy.^2,30,31^

Compared to previously described techniques, pulsed-field ablation (PFA) is a newer and less investigated energy source, promising safe and durable PVI, while protecting non-myocardial tissues in the atria, thereby reducing complication rates.^2,32–36^

The durability of PVI, besides non-PV triggers, has been reported to be the main factor affecting the AF-recurrence rates in patients undergoing PVI.^37^ However, PVI durability assessed by systematic remapping in all patients after a pre-specified follow-up has rarely been investigated in previous randomized controlled trials (RCT) in head-to-head comparisons of different ablation energies, especially since the advent of PFA^2,32,33,38^ or laser balloon.^30,31,39^ On the other hand, smaller studies presenting data of lower evidence levels than RCTs regarding lesion durability for RFA and cryoenergy are widely available.^3–15,17–20,30,40^ A systematic review of such studies, adjusting for important confounders is lacking. Using random-effects meta-analysis and meta-regression techniques, the present study aimed to systematically review the literature and offer a direct comparison of the durability of PVI using RFA, cryoballoon, laser, and PFA in patients undergoing remapping at a pre-specified timepoint during follow-up, regardless of AF recurrence.

## Methods

The results are presented according to the PRISMA statement on systematic reviews.^41^

### Data sources and search

We designed the search strategy together with the guidance of a research librarian. On 29 January 2023, we performed a database search in PubMed, MEDLINE, and Embase by combining synonyms of the terms ‘PVI’, ‘AF’, ‘catheter ablation’, ‘PFA’, and included abstracts published between January 2010 and January 2023. (See Supplementary material online, *Supplemental Appendix*)

### Study selection

Abstracts and studies were included if they followed pre-specified criteria: (i) the study was conducted in humans, (ii) was not a meta-analysis or review, (iii) evaluated patients with AF undergoing PVI with or without additional ablation lesions, (iv) did not evaluate surgical PVI, (v) examined a minimum of 10 patients, (vi) the entire population of the index procedure was studied in a second, mandatory electrophysiological study.

The selection, validation, and data extraction of the study were carried out by four independent researchers from the study team (TS, JdF, DM, and QA) in a dedicated RedCap database hosted at the University Hospital of Basel. More information on the selected studies and the extracted data is presented in the Supplementary material Online, *Supplemental Appendix*.

### Endpoints

The primary endpoint of our study was the number of veins durably isolated at follow-up depending on the ablation energy used.

Secondary endpoints were the proportion of patients with all veins durably isolated at follow-up depending on the energy used and the location of the veins persistently isolated at follow-up.

### Analysis of the lesion durability

Studies that evaluated patients who underwent PVI for AF were included if a second electrophysiological study had been performed. To be included, the study had to report the percentage of patients and/or PVs durably isolated at follow-up and clearly state the mean/median time to follow-up procedure. Studies that did not provide information on the time of remapping were excluded. The durability of the isolation was evaluated on a ‘per-patient’ (number of patients with all PV isolated) and ‘per-vein’ (number of veins isolated) analysis.

### Analysis of extra-pulmonary foci for atrial fibrillation recurrence

In all studies reporting the necessary data, we compared the rates of PV reconnection with the rates of AF recurrence. A higher percentage of patients with clinical AF recurrence than the percentage of patients with reconnected veins at remapping was interpreted as proof of extra-pulmonary AF foci.

### Evaluation of study quality

The quality of the included studies was assessed using the Newcastle Ottawa Scale (NOS) quality criteria and was graphically summarized.^42^ Complete information on study quality is presented in the Supplementary material Online, *Supplemental Appendix*.

### Statistical analysis

All analyses were performed using the R Statistical programme (R Foundation for Statistical Computing, Version 4.1.2 Vienna, Austria) by following the Cochrane Collaboration recommendations.^43^ The results are reported according to the Statement of Preferred Reporting Items for Systematic Reviews and Meta-Analysis (PRISMA)^41^ and the most recent guidelines.

All baseline characteristics of the patients were recorded as mean with standard deviation (SD) or as median and interquartile range (IQR). The median (IQR) was then converted into mean and SD to allow quantitative summaries as proposed in previous research.^44^

Data on the number of patients and veins durably isolated at follow-up were expressed as a percentage of the population/veins assessed in a second EP procedure, leading to the ‘per-vein’ or ‘per-patient’ analyses. Meta-analytic summary of the proportions of patients/veins that remained isolated at the follow-up procedure was calculated using the ‘metaprop’ function of the ‘meta’ package.^45^

To estimate the variability between studies, the risk estimates, and the confidence intervals, we used random effects models by inverse variance method, as proposed by the meta R package^45^ which accounts for intra- and inter-study variance. The variance between studies was calculated using the DerSimonian-Laird estimator, and the confidence interval of the tau-squared was calculated by using the Jackson method. Statistical heterogeneity was evaluated with the I^2^ statistic and an I^2^ > 50% indicates a high level of heterogeneity.

The percentages of isolated patients and/or veins were then compared between the studies and corrected for age, publication year, percentage of patients with paroxysmal or persistent AF, mean age of the cohort, percentage of women, and time to follow-up EP procedure using meta-regression.

Publication bias was evaluated using the funnel plot. Publication bias was evaluated using the New Ottawa Scale.^42^

The proportions of the various studies are presented graphically using Forrest plots with pooled proportions, 95% CI, weights of the study, and basic population characteristics.

A *P*-value less than or equal to 0.05 was considered statistically significant.

## Results

### Selected studies

Of the 3129 abstracts screened, 21 abstracts were excluded as they were not conducted in humans, 246 were reviews or meta-analyses, 638 did not focus on PVI for AF, 86 focused on surgical ablations, and 169 reported less than 10 patients and 1906 studies did not evaluate the durability of the lesion in a mandatory second electrophysiological study in the overall cohort. During a full-text review of the 63 remaining studies, six abstracts were further excluded because they did not provide the type of energy used and 11 because they were duplicated studies, leaving a total of 46 studies, out of which only 45 provided a follow-up duration. After reviewing the ablation outcomes, six studies were further excluded from the final analysis because they did not provide sufficient information on the number of veins reconnected at follow-up, and 17 for doing redo studies in only AF recurrence patients. Finally, three studies were excluded as only abstracts were published (see Supplementary material online, *Supplemental Appendix*). The final analysis included 19^3–8,10–13,15,17–20,30,32,33,40^ studies that investigated 1050 patients (mean age 60 years, 31% women).

### Ablation results at baseline

In all studies that provided the appropriate data, a weighted mean of 99.7% of all veins were isolated and 99.4% of all patients had all PV isolated during index ablation. When stratifying the results by the type of energy used and index procedure, PFA had the best index ablation results (100% veins and 100% isolated patients at baseline, two studies,^32,33^ 130 patients), while the worst were seen in the laser group (98% isolated veins, 95% isolated patients, one study,^30^ 56 patients). The two other energy sources showed a very high acute ablation success rate (RFA 99.8% veins, 99.9% patients, 13 studies,^3–8,10–13,15,17,40^ 910 patients and cryoballoon 99.5% veins, 98.3% patients, 4 studies^17–20^ 112 patients). A detailed overview of the weighted means in a per-vein and per-patient analysis stratified by the type of energy used is shown in *Table 1*.

**Table 1:** Results of pulmonary vein isolation at baseline and at follow-up

Energy type	Weighted mean of isolated veins at baseline	Weighted mean of isolated veins at follow-up
Cryoballoon	99.47	78.88
Radiofrequency	99.81	70.92
Laser	98.06	83.94
Pulsed-field ablation	100.00	87.18

Energy type	Weighted mean of patients with all PVs isolated at baseline	Weighted mean of patients with all PVs isolated at follow-up
Cryoballoon	98.25	53.57
Radiofrequency	99.86	45.81
Laser	94.64	61.54
Pulsed-field ablation	100.00	70.00

### Durability of ablation at follow-up—per-vein pooled analysis

In the follow-up EP study, which was conducted between 2 and 7 months, 75.5% of the veins remained durably isolated across all energy sources.

**Figure 1:**
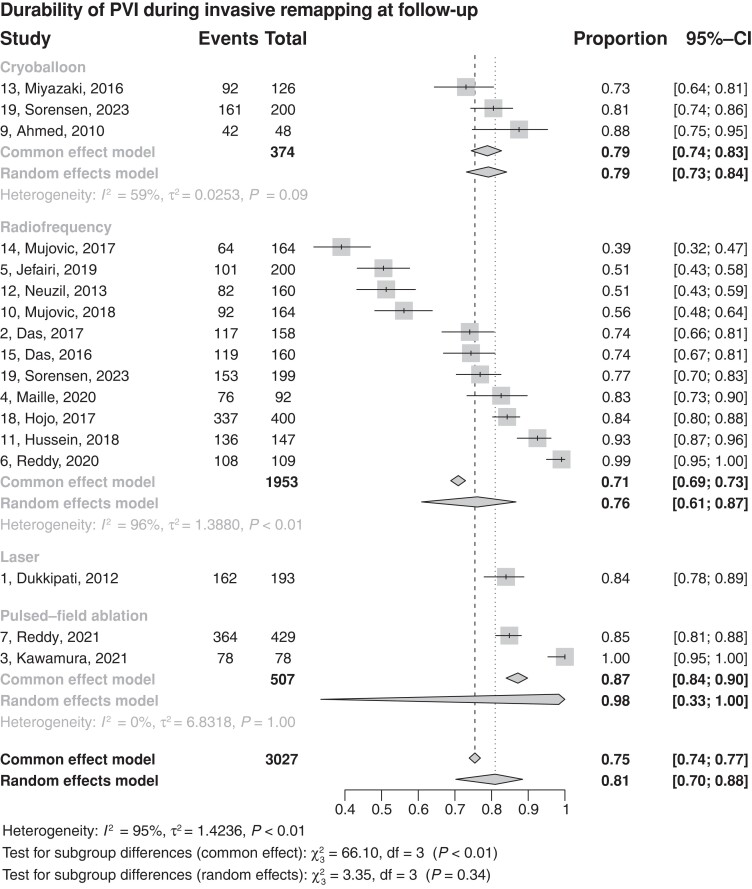
Forest plot of weighted means estimates in a per-vein analysis. CI, confidence interval; PVI, pulmonary vein isolation.

In a weighted analysis of the percentages of veins durably isolated during follow-up, RFA estimates were the lowest of all energy sources at 71% (95% CI 69–73, 11 studies^3–11,17,40^) slightly lower than cryoballoon (weighted mean 79%, 95%CI 74–83, three studies^17,19,20^). A higher percentage of veins durably isolated at follow-up was reported in those ablated with laser (weighted mean 84%, 95%CI 78–89, one study^30^) and PFA (87%, 95%CI 84–90, two studies ^32,33^). (*Table 1*, *Figure 1*).

### Durability of ablation at follow-up—per-patient pooled analysis

**Figure 2:**
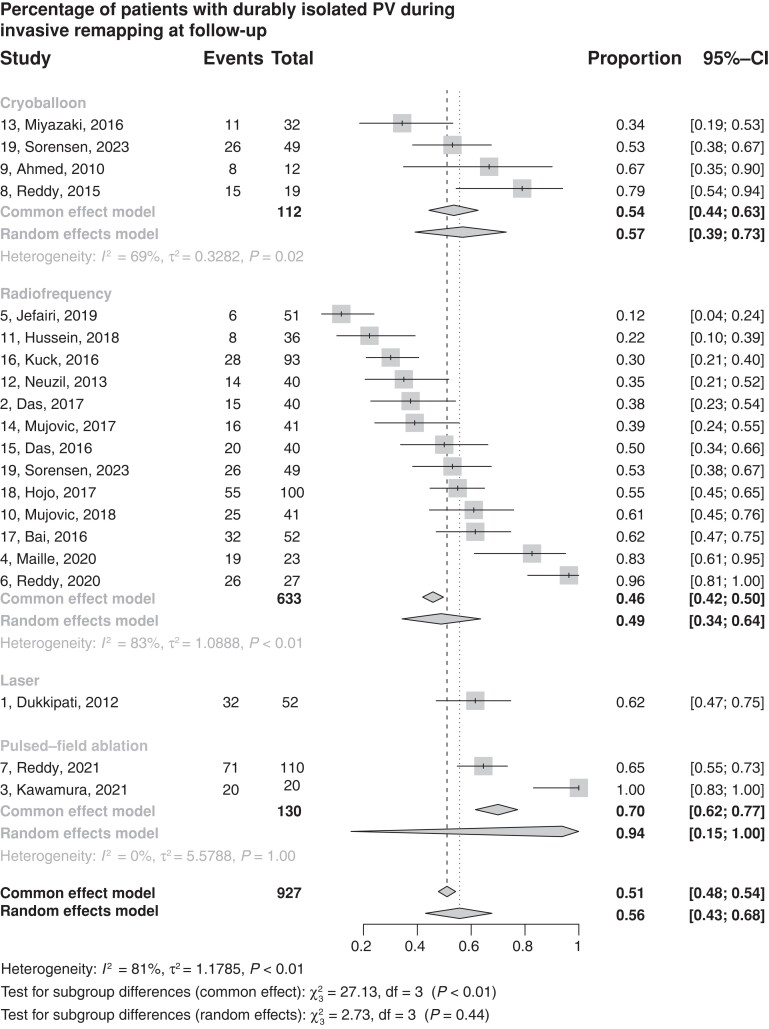
Forest plot of the estimated means of patients with all veins durably isolated at follow-up. CI, confidence interval; PV, pulmonary vein.

The percentage of patients who presented with all durably isolated PVs at follow-up was the lowest in RFA with a weighted estimate of 46% (95% CI 42–50%, 13 studies ^3–8,10–14,17,40^), then 54% (95%CI 44–63, 4 studies^17–20^) for cryoballoon, while in laser the weighted estimate was 62% (95%CI 47–75, one study^30^) and 70% (95% CI 62–77, two studies^32,33^) for PFA. (*Figure 2*).

### Pooled analysis of lesion durability depending on vein location

**Figure 3:**
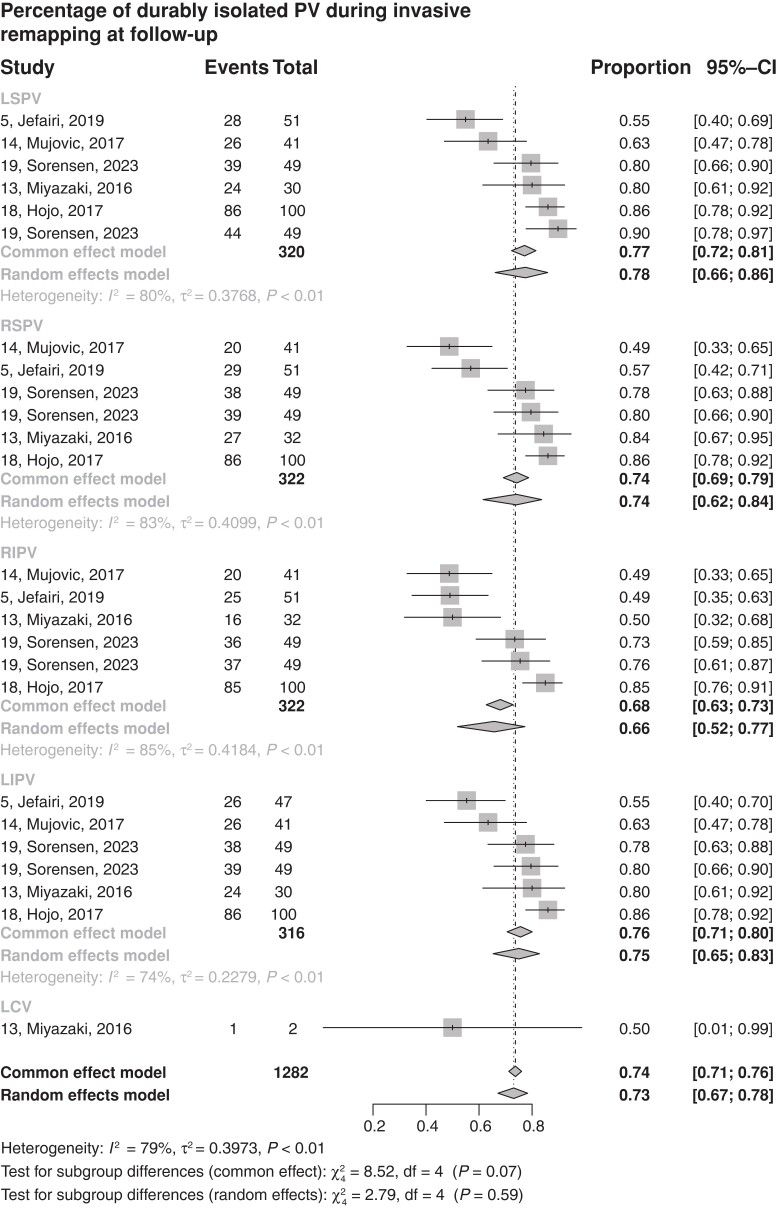
Estimated percentage of veins durably isolated at follow-up depending on the vein location and anatomy. CI, confidence interval, LCV, left common vein; LIPV, left inferior pulmonary vein; LSPV, left superior pulmonary vein; PV, pulmonary vein; RIPV, right inferior pulmonary vein; RSPV, right superior pulmonary vein.

When evaluating the durability of the lesion depending on the location of the vein, the left veins were more often durably isolated in the follow-up than the ones on the right: left superior pulmonary vein—LSPV 77% (95%CI 72–81, five studies^5,10,15,17,20^) and left inferior pulmonary vein—LIPV 76% (95%CI 71–80, five studies^5,10,15,17,20^) vs. right superior pulmonary vein—RSPV 74% (95%CI 69–79, five studies^5,10,15,17,20^) and right inferior pulmonary vein—RIPV 68% (95%CI 63–73, five studies.^5,10,15,17,20^). The one study^20^ reporting data in patients with a left common vein (LCV) reported low ablation durability (50%, 95%CI 1–99) (*Figure 3*).

### Meta-regression on a per-vein analysis and per-patient analysis

When investigating the durability of the lesion at follow-up on a per-vein analysis and adjusting for the time to follow-up EP study, publication year, mean age of the cohort, percentage of included women, and type of AF, none of the ablation energy sources were more likely to show durable isolation of PV during follow-up (*Table 2*). Similarly, no energy source was more likely to have higher percentages of patients with all PV persistently isolated at follow-up (see Supplementary material online, *Supplemental Appendix*).

**Table 2:** Meta-regression of energy type and covariables on the number of isolated veins at follow-up

Variable	Estimate	Lower CI	Upper CI	*P*-value
Energy type: Radiofrequency	0.64	0.14	3.01	0.57
Energy type: Laser	1.00	0.10	9.50	0.99
Energy type: Pulsed-field	1.07	0.06	17.93	0.96
Publication year	1.10	0.92	1.32	0.3
Mean age	0.72	0.51	1.01	0.06
% of women	1.04	0.97	1.11	0.23
% with hypertension	1.03	0.99	1.07	0.11
% of paroxysmal AF	0.98	0.96	1.00	0.07
Mean duration until redo	1.32	0.68	2.54	0.41

AF, atrial fibrillation; CI, confidence interval.

### Assessment of publication bias

**Figure 4:**
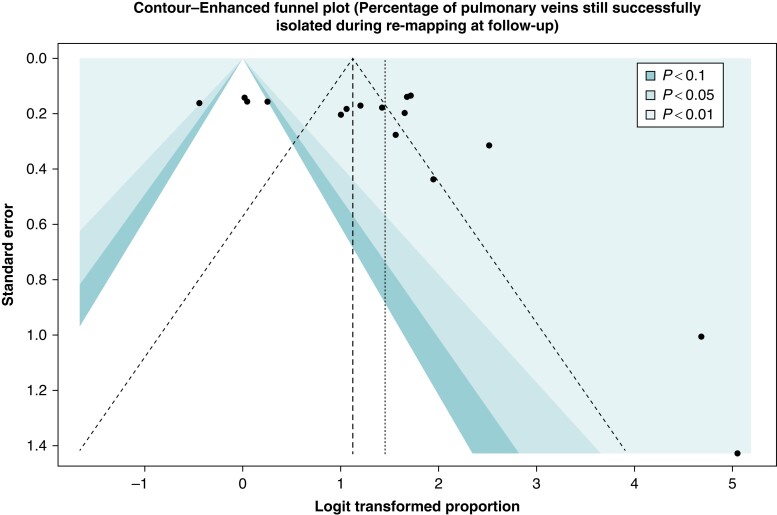
Evaluation of publication bias. The black and grey funnel represents the ideal study distribution (no publication bias), while the dotted funnel represents the actual study distribution. The farther away the dotted funnel from the ideal funnel is the larger the expected publication bias. The black points in the figure represent the included studies, the smaller the study, the larger the standard error.

The evaluation publication bias was evaluated for the percentage of PV durably isolated at follow-up procedures using the funnel plot, which showed a significant publication bias (*Figure 4*).

### Assessment of study quality

The quality of the study was evaluated using the New Ottawa Scale, evaluating the studies from 0 to 7 points depending on the number of criteria met. On average, the included studies were of moderate study quality (see Supplementary material online, *Supplemental Appendix*).

## Discussion

The aim of our systematic review and meta-analysis was to provide an up-to-date snapshot of the rapidly evolving literature on the durability of ablation lesions based on remapping studies after PVI, depending on the ablation energy used. We found no evidence that the durability of the PVI is significantly different between various ablation techniques after adjusting for age, sex, type of AF and time to follow-up EP study, both in a per-vein and in a per-patient analysis.

We also report several other important findings: (i) The durability of RFA lesion and cryoballoon were comparable on a per-vein analysis, with slightly increased estimates for cryoballoon (71% vs. 79%). Laser balloon and PFA had increased lesion durability in a pooled analysis, estimated at 84% and 87%, respectively. The number of studies evaluating these ablation energies,^30,32,33^ however, was significantly smaller than the ones evaluating RFA and cryoballoon (3 studies compared to 16 studies). (ii) The proportion of patients with all veins durably isolated at follow-up was similar between RFA, cryoballoon, and laser balloon, while the highest percentages were reported in the case of PFA. (iii) There were no significant associations between time to invasive remapping, age, and percentage of women included in the study in the percentage of PV or patients with all veins persistently isolated during follow-up. (iv) The ablation lesions of the RIPV appeared to be quantitatively less durable than the other PVs. (v) There seems to be a non-negligible role of non-PV AF foci reflected by the higher percentages of patients with AF recurrence than the percentage of patients with PV reconnection.

In this study, the lesion durability of RFA and cryoballoon ablation was found to be comparable after adjusting for other baseline population parameters in a per-vein analysis. As such, the choice of ablation energies should continue depending on the operator’s preference, the anatomy of the atria and the PVs, and the need for additional ablation lines.^46^ Due to its high versatility, it is possible that there is a risk of selection bias leading to poor reported RFA lesion durability, as patients with complex left atrial anatomy will be usually ablated with RFA. For this reason, we believe that RFA will probably continue to see wide use due to its versatility with the possibility of performing additional ablation lines without requiring an additional ablation catheter. At the same time, RFA and cryoballoon catheters are continually improving, and lesion persistency is expected to increase in the coming years.^1,15,47–51^ Only one study that evaluated laser balloon ablation was included in this analysis, as such a conclusion cannot be drawn regarding this ablation energy.^2^ More studies comparing laser balloon ablation to cryoballoon of RFA are warranted. Newer methods, like PFA, promise a more durable PVI.^34^ Despite such assumptions being validated in our study, we find that current data on these techniques is limited, and the studies included in the current meta-analysis^32,33^ had a relatively short time to follow up. More research is needed to provide an accurate estimate of their mid- and long-term efficacy.

Due to the heterogeneity of the evaluated studies regarding publication year, methods used, available technology at the time, as well as differences in baseline characteristics, a direct comparison of the findings is difficult. For this reason, we used meta-regression techniques to adjust for various parameters to make the ablation energies more comparable. We report that the current data is not able to offer convincing proof that any of the assessed techniques are superior. We found no significant associations between sex, age, or time to follow-up and the number of PV durably isolated or the number of patients with all PV persistently isolated.

The right PVs, particularly the RIPV, are less likely to be durably isolated at follow-up compared to the left-sided veins. A possible explanation for this finding may be related to the anatomical proximity of the location of the transseptal puncture and the ostium of the right PVs, which requires acute angles of the ablation catheter. Such procedural difficulties might decrease contact between the catheter tip and the tissue, potentially leading to less durable isolation in the case of thermal ablation modalities. A low durability was reported for ablation of LCV in one study using cryoballoon,^20^ potentially favouring the use of other energy modalities with this anatomy.

When evaluating AF recurrence, we found that 4/11 studies^4,7,13,30^ reported higher percentages of patients with AF recurrence than percentages of patients with PV reconnection, indicating extra-PV AF foci responsible for AF recurrence (see Supplementary material Online, *Supplemental Appendix*). The mechanisms of such foci and their role in AF recurrence need further investigation.

Our study has several limitations: (i) Despite the relatively large number of studies included in the analysis, the number of patients evaluated in each study is small, with significant heterogeneity, publication bias, and moderate study quality. Only three small RCT met the inclusion criteria (PRESSURE Trial—early PVI redo procedure regardless of symptoms vs. standard care^3^; Gap-AF–AFNET 1—impact of complete vs. incomplete circumferential PVI^12^ and Sorensen et al—investigation gap location in RFA vs. cryoballoon ablation^17^). (ii) Several studies had to be excluded due to incomplete data reporting. (iii) Only a small number of studies reported patients ablated with laser balloon and PFA, which affected the accuracy of the reported results for these types of energy. (iv) Due to the low number of studies published we were unable to perform a network-type analysis. (v) Due to the different catheters used in the included studies, it may be difficult to generalize the results for a given energy source.

## Conclusions

We observed no significant difference in the durability of the ablation lesions of the four evaluated energies after adjusting for procedural and baseline populational characteristics. RFA and cryoballoon offer similar durability of PVI lesions while newer ablation techniques show promising results with numerically higher PVI durability in recent studies which require validation through further larger studies. The RIPV is the most often reconnected PV.

## Supplementary Material

euad335_Supplementary_Data

## Data Availability

All relevant data are within the manuscript and its supporting Information files.
