# Supplementary material for: Durability of pulmonary vein isolation for atrial fibrillation: a meta-analysis and systematic review
Source: Europace. 2023 Nov 7;25(11):euad335. doi: 10.1093/europace/euad335 (PMC10664405; doi:10.1093/europace/euad335)
Supplement: euad335_Supplementary_Data [file euad335_supplementary_data.docx]

Supplemental Appendix

Table of Contents

[Supplement Figure 1 : Flow chart illustrating the selection process of the studies. 2](#_Toc140423432)

[Supplement Table 1: Overview of the type of study, number of patients recruited and number of patients analysed in each included study 3](#_Toc140423433)

[Supplement Table 2: Baseline characteristics and type of energy used for ablation in each included study 8](#_Toc140423434)

[Supplement Table 3: Overview of studies reporting recurrence rates and the percentage of patients with all pulmonary veins durably isolated at follow-up 18](#_Toc140423435)

[Supplement Table 4: Metaregression of energy type and covariables on the number of patients with all isolated vein at time of follow-up 21](#_Toc140423436)

[Supplement Figure 2: Evaluation of the study quality according to the New Ottawa Scale 22](#_Toc140423437)

## Supplement Figure 1 : Flow chart illustrating the selection process of the studies. Abbreviations: AF-atrial fibrillation, PVI – pulmonary vein isolation

## Supplement Table 1: Overview of the type of study, number of patients recruited and number of patients analysed in each included study

| **record_id** | **PVI-Meta ID** | **Main author** | **Abstract** | **Study begin** | **Study end** | **Study duration** | **Typoe of study** | **Country** | **Centers** | **Nr. recruited** | **Nr. analyzed** | **Re-analysis of another trial?** | **If reanlaysis, which trial?** |
| --- | --- | --- | --- | --- | --- | --- | --- | --- | --- | --- | --- | --- | --- |
| 55 | 1 | Dukkipati | full study |  |  |  | Retrospective: Cohort | international | Multicentric | 56 | 56 | Yes | UNK |
| 152 | 2 | Das | full study |  |  |  | RCT single-blind | United Kingdom | Monocentric | 83 | 80 | No |  |
| 182 | 3 | Kawamura | full study | 2018-01-20 | 2020-12-21 | 2.9 | Retrospective: Cohort | international | Multicentric | 45 | 20 | Yes | PEFCAT |
| 198 | 4 | Maille | full study |  |  |  | Retrospective: Cohort | United Kingdom | Unknown | 23 | 23 | No |  |
| 221 | 5 | Jefairi | full study | 2013-06-01 | 2015-03-01 | 1.7 | Prospective: Cohort | France | Monocentric | 60 | 51 | No |  |
| 235 | 6 | Reddy | full study | 2018-05-01 | 2019-05-01 | 1.0 | Prospective: Cohort | international | Multicentric | 65 | 65 | No |  |
| 247 | 7 | Reddy | full study |  |  |  | Prospective: Cohort | international | Multicentric | 121 | 121 | No |  |
| 250 | 8 | Reddy | full study | 2012-07-01 | 2012-11-01 | 0.3 | Prospective: Cohort | Czech Republic | Monocentric | 21 | 21 | Yes | SUPIR |
| 257 | 9 | Ahmed | full study |  |  |  | Prospective: Cohort | Czech Republic | Monocentric | 12 | 12 | No |  |
| 307 | 10 | Mujovic | full study | 2015-11-01 | 2016-02-01 | 0.3 | Retrospective: Cohort | Serbia | Monocentric | 41 | 41 | No |  |
| 330 | 11 | Hussein | full study | 2015-01-01 | 2018-04-01 | 3.2 | Prospective: Cohort | international | Multicentric | 40 | 40 | No |  |
| 348 | 12 | Neuzil | full study |  |  |  | Prospective: Cohort | international | Multicentric | 46 | 46 | Yes | EFFICAS I |
| 402 | 13 | Miyazaki | full study |  |  |  | Prospective: Cohort | Japan | Monocentric | 32 | 32 | No |  |
| 419 | 14 | Mujovic | full study | 2015-10-01 | 2016-05-01 | 0.6 | Prospective: Cohort | Serbia | Monocentric | 42 | 41 | No |  |
| 428 | 15 | Das | full study |  |  |  | Prospective: Cohort | United Kingdom | Monocentric | 40 | 40 | Yes | PRESSURE |
| 772 | 16 | Kuck | full study | 2006-02-01 | 2010-08-01 | 4.5 | RCT open labelled | Germany | Multicentric | 233 | 233 | No |  |
| 879 | 17 | Bai | full study | 2010-10-01 | 2011-07-01 | 0.7 | Prospective: Cohort | China | Unknown | 52 | 52 | No |  |
| 1,824 | 18 | Hojo | full study | 2013-08-01 | 2015-09-01 | 2.1 | Prospective: Cohort | Japan | Monocentric | 141 | 100 | No |  |
| 2,956 | 19 | Sorensen | full study | 2015-06-01 | 2018-08-01 | 3.2 | RCT open labelled | Denmark | Monocentric | 98 | 98 | Yes | RACE‐AF Trial |

Abbreviations: PVI- pulmonary vein isolation, RCT – randomized controlled trial, UNK – unknown,

## Supplement Table 2: Baseline characteristics and type of energy used for ablation in each included study

| **PVI-Meta ID** | **Study info** | **Main cohort or subgroup** | **Group meta-analyzed** | **Age** | **% of women** | **LVEF** | **% of PAF patients** | **% of persAF patients** | **Type of energy used for the ablation** |
| --- | --- | --- | --- | --- | --- | --- | --- | --- | --- |
| 1 | 1, Dukkipati, 2012 | Overall cohort | 1 | 57 (10) | 28.6 |  | 100.0 | 0.0 | laser |
| 2 | 2, Das, 2017 | Overall cohort | 1 | 62 (3) | 47.5 |  | 100.0 | 0.0 | rf |
| 2 | 2, Das, 2017 | Standard Care | 0 | 63 (3) | 55.0 |  | 100.0 | 0.0 | rf |
| 2 | 2, Das, 2017 | Repeat Study | 0 | 52 (11) | 40.0 |  | 100.0 | 0.0 | rf |
| 3 | 3, Kawamura, 2021 | Overall cohort | 1 | 56 (12) | 25.0 | 64 (4) | 100.0 | 0.0 | pfa |
| 4 | 4, Maille, 2020 | Overall cohort | 1 | 60 (6) | 26.1 |  | 0.0 | 100.0 | rf |
| 4 | 4, Maille, 2020 | Volume reverse remodeling: no | 0 | 60 (3) | 38.5 |  | 0.0 | 100.0 | rf |
| 4 | 4, Maille, 2020 | Volume reverse remodeling: yes | 0 | 61 (2) | 10.0 |  | 0.0 | 100.0 | rf |
| 4 | 4, Maille, 2020 | Conduction reverse remodeling: no | 0 | 59 (2) | 16.7 |  | 0.0 | 100.0 | rf |
| 4 | 4, Maille, 2020 | Conduction reverse remodeling: yes | 0 | 62 (3) | 29.4 |  | 0.0 | 100.0 | rf |
| 4 | 4, Maille, 2020 | Voltage reverse remodeling: no | 0 | 59 (2) | 20.0 |  | 0.0 | 100.0 | rf |
| 4 | 4, Maille, 2020 | Voltage reverse remodeling: yes | 0 | 64 (2) | 50.0 |  | 0.0 | 100.0 | rf |
| 5 | 5, Jefairi, 2019 | Overall cohort | 1 | 61 (9) | 23.5 | 63 (7) | 100.0 | 0.0 | rf |
| 5 | 5, Jefairi, 2019 | Conventional | 0 | 60 (8) | 35.7 | 61 (7) | 100.0 | 0.0 | rf |
| 5 | 5, Jefairi, 2019 | Circular | 0 | 62 (9) | 8.7 | 65 (5) | 100.0 | 0.0 | rf |
| 6 | 6, Reddy, 2020 | Overall cohort | 1 | 62 (9) | 32.3 | 60 (7) | 61.5 | 38.5 | rf |
| 6 | 6, Reddy, 2020 | Remap cohort | 0 | 65 (10) | 29.6 | 59 (9) | 59.3 | 44.4 | rf |
| 7 | 7, Reddy, 2021 | Overall cohort | 1 | 57 (10) | 26.4 | 62 (6) | 100.0 | 0.0 | pfa |
| 8 | 8, Reddy, 2015 | Overall cohort | 1 | 60 (11) | 57.1 |  | 100.0 | 0.0 | cb |
| 9 | 9, Ahmed, 2010 | Overall cohort | 1 | 54 (11) | 25.0 | 68 (8) | 100.0 | 0.0 | cb |
| 10 | 10, Mujovic, 2018 | Overall cohort | 1 | 60 (8) | 22.0 | 60 (2) | 56.1 | 43.9 | rf |
| 10 | 10, Mujovic, 2018 | Early recurrence of atrial fibrillation | 0 | 62 (7) | 17.6 | 60 (2) | 58.8 | 41.2 | rf |
| 10 | 10, Mujovic, 2018 | No early recurrence of atrial fibrillation | 0 | 58 (9) | 25.0 | 60 (2) | 54.2 | 45.8 | rf |
| 11 | 11, Hussein, 2018 | Overall cohort | 1 | 61 (8) | 25.0 |  | 0.0 | 100.0 | rf |
| 11 | 11, Hussein, 2018 | No recurrence of atrial tachycardia | 0 | 62 (3) | 25.0 |  | 0.0 | 100.0 | rf |
| 11 | 11, Hussein, 2018 | Recurrence of atrial tachycardia | 0 | 57 (5) | 25.0 |  | 0.0 | 100.0 | rf |
| 12 | 12, Neuzil, 2013 | Cohort with follow-up | 1 | 59 (10) |  |  | 97.5 | 2.5 | rf |
| 13 | 13, Miyazaki, 2016 | Overall cohort | 1 | 65 (9) | 31.2 | 66 (5) | 100.0 | 0.0 | cb |
| 14 | 14, Mujovic, 2017 | Overall cohort | 1 | 59 (8) | 22.0 |  | 56.1 | 43.9 | rf |
| 15 | 15, Das, 2016 | Overall cohort | 1 | 58 (12) | 40.0 |  | 100.0 | 0.0 | rf |
| 15 | 15, Das, 2016 | Acute pulmonary vein reconnection | 0 | 58 (10) | 36.4 |  | 100.0 | 0.0 | rf |
| 15 | 15, Das, 2016 | No acute pulmonary vein reconnection | 0 | 59 (14) | 44.4 |  | 100.0 | 0.0 | rf |
| 15 | 15, Das, 2016 | Late pulmonary vein reconnection | 0 | 57 (12) | 40.0 |  | 100.0 | 0.0 | rf |
| 15 | 15, Das, 2016 | No late pulmonary vein reconnection | 0 | 60 (11) | 40.0 |  | 100.0 | 0.0 | rf |
| 16 | 16, Kuck, 2016 | Overall cohort | 1 | 62 (7) | 47.9 |  | 100.0 | 0.0 | rf |
| 17 | 17, Bai, 2016 | Overall cohort | 1 | 64 (10) | 17.3 | 56 (10) | 0.0 | 100.0 | rf |
| 17 | 17, Bai, 2016 | Pulmonary vein antral isolation | 0 | 63 (11) | 20.0 | 55 (12) | 0.0 | 100.0 | rf |
| 17 | 17, Bai, 2016 | Pulmonary vein antral isolation + left atrial posterior wall ablation | 0 | 64 (10) | 15.6 | 57 (9) | 0.0 | 100.0 | rf |
| 18 | 18, Hojo, 2017 | Overall cohort | 1 | 63 (10) | 29.0 | 63 (11) | 87.0 | 13.0 | rf |
| 18 | 18, Hojo, 2017 | Non - obstructive sleep apnea | 0 | 60 (12) | 33.3 | 63 (7) | 89.4 | 10.6 | rf |
| 18 | 18, Hojo, 2017 | Treated obstructive sleep apnea | 0 | 66 (10) | 27.3 | 64 (10) | 72.7 | 27.3 | rf |
| 18 | 18, Hojo, 2017 | Obstructive sleep apnea | 0 | 68 (9) | 17.4 | 62 (14) | 87.0 | 13.0 | rf |
| 19 | 19, Sorensen, 2023 | Radiofrequency group | 1 | 62 (4) | 28.6 |  | 100.0 | 0.0 | rf |
| 19 | 19, Sorensen, 2023 | Cryoballoon group | 1 | 60 (2) | 34.7 |  | 100.0 | 0.0 | cb |
| 19 | 19, Sorensen, 2023 | Overall cohort | 0 | 61 (3) | 31.6 |  | 100.0 | 0.0 | >1 energy |

## Abbreviations: cb- cryoballoon, LVEF – left ventricular ejection fraction, PVI – pulmonary vein isolation, LVEF – left ventricular ejection fraction, PAF – paroxysmal atrial fibrillation, persAF – persistent atrial fibrillation, pfa- pulsed field ablation, rf-radiofrequency,Supplement Table 3: Overview of studies reporting recurrence rates and the percentage of patients with all pulmonary veins durably isolated at follow-up

| **Study info** | **Energy** | **Patient followed-up and remapped/ablated** | **Nr. clinical recurrence** | **% clinical recurrence** | **Nr. patients with reconnected veins** | **% patients with reconnected veins** |
| --- | --- | --- | --- | --- | --- | --- |
| 1, Dukkipati, 2012 | Laser | 52 | 37 | 71.2 | 32 | 38.5 |
| 4, Maille, 2020 | Radiofrequency | 23 | 5 | 21.7 | 19 | 17.4 |
| 5, Jefairi, 2019 | Radiofrequency | 51 | 10 | 19.6 | 6 | 88.2 |
| 8, Reddy, 2015 | Cryoballoon | 19 | 2 | 10.5 | 15 | 21.1 |
| 10, Mujovic, 2018 | Radiofrequency | 41 | 17 | 41.5 | 25 | 39.0 |
| 13, Miyazaki, 2016 | Cryoballoon | 32 | 8 | 25.0 | 11 | 65.6 |
| 14, Mujovic, 2017 | Radiofrequency | 41 | 2 | 4.9 | 16 | 61.0 |
| 15, Das, 2016 | Radiofrequency | 40 | 8 | 20.0 | 20 | 50.0 |
| 16, Kuck, 2016 | Radiofrequency | 93 | 56 | 60.2 | 28 | 69.9 |
| 17, Bai, 2016 | Radiofrequency | 52 | 37 | 71.2 | 32 | 38.5 |
| 18, Hojo, 2017 | Radiofrequency | 100 | 24 | 24.0 | 55 | 45.0 |

Blue → % clinical recurrence > % patients with reconnected veins → extra-pulmonary foci?

Red → % clinical recurrence < % patients with reconnected veins → non-arrhythmogenic reconnection?

## Supplement Table 4: Metaregression of energy type and covariables on the number of patients with all isolated veins at time of follow-up

|  | Estimate | lower CI | upper CI | p-value |
| --- | --- | --- | --- | --- |
| Energy type: Radiofrequency | 0.63 | 0.11 | 3.61 | 0.60440 |
| Energy type: Laser | 1.64 | 0.15 | 18.38 | 0.68652 |
| Energy type: Pulsed-field | 7.91 | 0.37 | 168.35 | 0.18501 |
| Publication year | 0.99 | 0.81 | 1.22 | 0.95741 |
| mean age | 0.96 | 0.63 | 1.46 | 0.85391 |
| % of women | 1.07 | 0.97 | 1.19 | 0.18102 |
| % with hypertension | 1.00 | 0.96 | 1.04 | 0.88616 |
| % of paroxysmal AF | 0.98 | 0.96 | 1.00 | 0.11868 |
| mean duration until redo | 1.16 | 0.55 | 2.43 | 0.70034 |

## Supplement Figure 2: Evaluation of the study quality according to the New Ottawa Scale. Abbreviations : NOS – New Ottawa Scale, RCT – randomized controlled trial
